# Design-Driven Gel-Based Delivery Systems for Bioactives in Sports Nutrition

**DOI:** 10.3390/gels12060525

**Published:** 2026-06-11

**Authors:** Yien Xiang, Fan Yao, Xin Jin, Qiao Li, Jianwei Zang, Jun Wu

**Affiliations:** 1 Jilin University Engineering Laboratory for Translational Medicine of Hepatobiliary and Pancreatic Diseases, The Second Norman Bethune Clinical Medical College of Jilin University, Changchun 130021, China; xiangyien@jlu.edu.cn (Y.X.); liqiao362@163.com (Q.L.); 2 Changchun University of Chinese Medicine, Changchun 130117, China; 13258841878@163.com (F.Y.); kim37432131@163.com (X.J.); 3 Sanya Institute of Nanjing Agricultural University, College of Food Science and Technology, Nanjing Agricultural University, Nanjing 210095, China; zangjv@yeah.net

**Keywords:** bioactive compounds, delivery systems, nutrition, gel-based food delivery systems

## Abstract

Sports nutrition products are increasingly expected to deliver bioactive compounds that aid in recovery, reduce fatigue, and support physiological regulation, going beyond merely providing energy and nutrients. However, many bioactive compounds face challenges such as poor aqueous dispersibility, limited stability, low bioaccessibility, or inefficient absorption, which hinder their practical use in real food products. This review critically examines food-grade, gel-based delivery systems for bioactive compounds in sports nutrition from a design-driven perspective. It focuses on hydrogels, microgels, emulsion gels, protein gel matrices, and multicomponent gel architectures that prioritize structural stability, digestion-triggered responsiveness, and compatibility with food. Key design principles are discussed, including the need to maintain stability during processing and storage, balance protection with release, and tailor delivery structures to sports-specific constraints such as gastrointestinal tolerance, osmotic load, nutrient timing, and changes in digestion related to exercise. The review also analyzes the effectiveness of gel-based and hybrid systems in liquid, solid, and semi-solid sports nutrition products, emphasizing how the product format and consumption scenario can influence delivery performance. A design decision framework is proposed to align bioactive properties, food format, target release profile, and exercise-stage requirements with appropriate delivery architectures. Current challenges are also addressed, including difficulties in predicting structure–function relationships, limited robustness during scale-up processes, and inadequate functional evaluation. Overall, gel-based food delivery systems provide a promising solution for improving the stability, release behavior, and practical functionality of bioactives in sports nutrition.

## 1. Introduction

In recent years, increasing global health awareness, changing lifestyles, and a rising public interest in physical exercise have significantly boosted the consumption of nutritional and functional foods [1]. Food is no longer viewed merely as a means to meet basic nutritional needs; it is increasingly recognized for its roles in promoting health, enhancing physiological function, and supporting active lifestyles. Growing consumer interest in health, natural ingredients, functionality, and personalized nutrition has propelled dietary supplements and sports nutrition products to become some of the fastest-growing segments in the food industry [2]. Reports indicate that, alongside better access to health-related information and rising nutritional literacy, the significance of food in health promotion has gained prominence, leading to the transition of sports nutrition from a niche market to broader consumer adoption [3,4].

Against this backdrop, functional foods aimed at athletes demonstrate considerable market potential. Compared to the general population, individuals who regularly engage in physical exercise have specific and dynamically changing needs regarding energy metabolism, nutrient turnover, and physiological recovery [5,6]. During exercise, energy expenditure significantly increases, nutrients are depleted quickly, and physiological stress intensifies. Inadequate or untimely nutritional supplementation can lead to accumulated fatigue, reduced athletic performance, and even long-term health risks [7,8]. It is important to note that high-intensity exercise training not only stresses skeletal muscle and the cardiovascular system but also affects liver metabolism. The oxidative stress, inflammatory responses, and drastic fluctuations in energy metabolism during exercise can increase the metabolic burden on the liver and impair hepatocyte function. Therefore, functional foods designed for athletes, which include active ingredients with antioxidant, anti-inflammatory, and energy metabolism-regulating properties, may help protect the liver and maintain its metabolic homeostasis. For patients with chronic liver disease, specialized delivery of branched-chain amino acids (BCAAs) and silymarin has been shown to enhance liver function and nutritional status. Consequently, functional foods are widely viewed as an effective means of supporting athletic performance, promoting recovery, and maintaining overall health [9,10]. As physical exercise expands from professional competition to general fitness and daily health management, the consumer base for functional foods continues to grow, solidifying their position within the food and nutrition industry [11].

It is also essential to note that functional foods are not limited to healthy individuals or athletes. Increasing attention is being given to those with heightened metabolic or physiological demands, including individuals requiring specialized nutritional support during recovery or periods of metabolic stress [12,13]. In these contexts, functional foods are expected to not only provide energy and essential nutrients but also to deliver bioactive compounds that support physiological regulation and metabolic balance [14]. This emerging demand underscores the importance of designing food systems that can efficiently stabilize, protect, and deliver functional ingredients within complex physiological environments.

Although the functional food market continues to grow, many existing products primarily focus on energy supplementation and post-exercise recovery. Most commercially available functional foods emphasize macronutrients—carbohydrates, proteins, and fats—to meet energy and nutritional needs during exercise [15,16]. In contrast, the systematic integration of bioactive functional factors, which have specific physiological regulatory effects such as antioxidant and anti-inflammatory properties, joint protection, and muscle mass improvement, remains relatively limited in functional foods [17,18]. Previous reviews have categorized the nutritional components in functional foods into basic nutrients and functional factors. It has been noted that functional factors have significant potential for improving physiological functions and supporting long-term exercise health; however, their development and application in actual products are still insufficient [12,19]. In this review, bioactive compounds primarily refer to food-derived functional ingredients that exert physiological effects beyond basic nutritional or energy supply. This includes polyphenols, carotenoids, omega-3 fatty acids, bioactive peptides, and selected sports-related ergogenic compounds like caffeine, creatine, β-hydroxy β-methylbutyrate, and β-alanine. Conventional carbohydrates and macronutrients, including glucose, fructose, sucrose, maltodextrin, proteins, and lipids, are discussed as nutritional or formulation components rather than bioactive compounds.

Furthermore, functional foods come in various physical forms, including beverages, solid and semi-solid foods, and nutritional supplements. Each form imposes distinct requirements on ingredient stability, processing adaptability, and bioavailability [11,20]. However, existing research has mainly focused on the types of nutritional components and their biological functions, with relatively little attention paid to stabilizing these active ingredients within real food matrices and ensuring their efficient release and absorption during digestion. Based on a structured literature survey covering food-grade delivery systems, gel-based architectures, digestion-responsive materials, and sports nutrition applications published primarily between 2006 and 2026, this review adopts a design-driven perspective to critically examine food-grade delivery systems for bioactive compounds in functional and sports nutrition applications. It places particular emphasis on the relationships among structural stability, digestion-triggered responsiveness, formulation constraints, and practical functionality within real food matrices. Additionally, this review discusses emerging gel-based and multicomponent delivery architectures, aiming to clarify how structural design influences nutrient protection, release behavior, gastrointestinal tolerance, and practical applicability under realistic sports nutrition conditions. Detailed search strategies, literature selection criteria, and the corresponding flowchart are provided in the Supplementary Materials.

## 2. Core Design Principles of Food-Grade Delivery Systems

**Figure 1:**
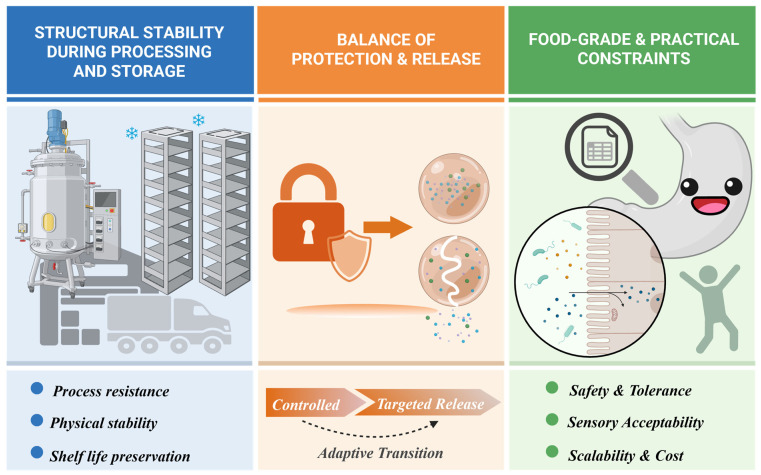
Core design principles of food-grade delivery systems for functional foods applications. Left: Structural stability during processing and storage, highlighting resistance to thermal treatment, mechanical shear, transport, and shelf-life stresses to preserve physical integrity and effective bioactive dosage. Center: Balance between protection and release, emphasizing an adaptive transition from protective encapsulation during processing and storage to controlled, digestion-triggered release, enabling timely nutrient availability. Right: Food-grade and practical constraints, including safety and gastrointestinal tolerance, sensory acceptability, scalability, and cost efficiency, which collectively determine real-world applicability.

In the field of functional foods, delivery systems are not standalone components; instead, they are incorporated within complex food matrices and dynamic gastrointestinal environments [21]. As a result, the design principles for these systems cannot be directly adapted from pharmaceutical delivery systems, which often emphasize “maximum protection” or “precise release.” In contrast, food-grade delivery systems for functional foods must strike a balance among processing feasibility, sensory compatibility, and digestion behavior [22]. To provide a design-oriented perspective, the following sections focus on three core functional design principles: (i) maintaining structural stability during processing and storage, (ii) balancing the inherent trade-off between protection and release, and (iii) addressing specific practical constraints related to food-grade and sports nutrition (as shown in Figure 1). This framework highlights how structural design choices can influence functional outcomes in realistic sports nutrition applications, rather than solely concentrating on the origin of the ingredients.

### 2.1. Structural Stability During Processing and Storage

Functional foods are often developed as liquid beverages, semi-solid gels, or ready-to-consume supplements. During their industrial production and usage, these products typically undergo various processes, including thermal treatments, high-shear homogenization, mixing, and dispersion, along with defined storage periods [23,24]. Throughout these stages, delivery systems experience physicochemical changes due to fluctuations in temperature, pH, and ionic strength. They also operate within complex formulations that can have high osmotic pressure and contain multiple components. Under these conditions, it is essential for delivery structures to maintain sufficient stability to prevent issues such as sedimentation, aggregation, or the chemical degradation of bioactive compounds [25,26]. Previous studies have shown that the physical stability of these structures during processing and storage is vital for ensuring that an effective dose of bioactive ingredients is retained in final food products [27,28].

From a design perspective, structural stability should not merely be seen as resistance to physical disruption but as the ability of a delivery system to maintain its functional integrity throughout processing, storage, and digestion. In practical food systems, processes such as homogenization, pumping, and mechanical agitation introduce significant shear forces and interfacial changes that can alter particle size distribution, interfacial composition, and internal network architecture [29]. Accumulating evidence suggests that structural attributes like particle size, interfacial organization, and internal phase structure play crucial roles in resisting aggregation and phase separation during both processing and storage [30]. Therefore, structural stability in functional foods should be viewed as a dynamic balance between maintaining stability during industrial processing and preserving adaptability for subsequent digestion.

However, maximum structural rigidity does not always equate to optimal nutritional functionality. Delivery systems that are overly compact or highly crosslinked may have excellent storage stability but can hinder enzyme accessibility, digestive breakdown, and intestinal bioaccessibility of the encapsulated compounds [31]. Arranz et al. [32] emphasized that ideal food delivery systems should undergo controlled structural changes or disintegration within the gastrointestinal tract. This approach facilitates the release of bioactive compounds at appropriate stages of digestion, rather than keeping them confined within rigid structures throughout their lifecycle. It is important to note that structural robustness and encapsulation efficiency do not always correlate positively with functional performance. Dense interfacial layers and compact internal networks may prevent oxidation, aggregation, or premature leakage during storage but can also delay nutrient release and reduce digestibility [33].

Recent studies further suggest that structural stability can be linked to specific formulation parameters rather than being solely a qualitative attribute. For example, in oat protein isolate/high-methoxyl pectin Pickering systems, complex particles with sizes ranging from 125.7 to 297.6 nm formed dense interfacial films, and the optimized formulation achieved an encapsulation efficiency of 85.63% while improving curcumin stability and controlling gastrointestinal release [34]. Similarly, in pea protein isolate gels, the addition of 0.75% cellulose nanocrystals produced the most compact network structure, increasing gel strength by 391.68% and water-holding capacity by 11.65%. However, further increases in nanocrystal concentration led to reduced structural benefits due to particle aggregation [35]. For hydrogel-based delivery systems, formulation-dependent effects have also been reported. Gelatin–genipin hydrogels containing curcumin nanoparticles exhibited substantially improved protection and release performance, with storage stability increasing by 174% compared to nanoparticle dispersions alone. This demonstrates that appropriate network reinforcement can markedly enhance functional retention [36]. Collectively, these studies indicate that effective delivery system design is often achieved within an optimal structural window rather than through maximizing particle reduction, crosslinking density, or gel rigidity. While excessively dense structures may improve storage stability, they can simultaneously hinder digestive disassembly and nutrient release. Conversely, insufficiently structured systems may fail to provide adequate protection during processing and storage. Therefore, current delivery system designs increasingly emphasize “functional retention” rather than simply maximizing encapsulation, particularly in sports nutrition contexts where nutrient availability is often time-sensitive.

From a processing feasibility perspective, the creation of many food-grade delivery structures is closely linked to industrial operations. Processes such as extrusion, homogenization, and shaping are not only central to food manufacturing but also critical for structuring delivery systems [37]. Research has demonstrated that by carefully adjusting processing conditions and the rheological properties of materials, it is possible to create delivery systems with adequate structural stability without resorting to complex chemical modifications. This offers practical avenues for their application in sports nutrition [38]. As a result, industrial scalability and structural robustness under real processing conditions are increasingly recognized as essential design considerations, along with delivery efficiency itself.

Overall, structural stability in sports nutrition delivery systems should not be oversimplified to mean that “more stability is better.” Instead, it presents a design challenge that necessitates careful trade-offs between processing robustness, storage stability, and digestive disassembly. One of the central challenges in creating delivery systems for sports nutrition is to maintain sufficient structural adaptability for downstream release while ensuring stability during industrial processing.

### 2.2. Design Trade-Off Between Protection and Release

In functional food applications, delivery systems must transition from a state of “protection” to one of “release.” This creates a significant trade-off between these two functions. Protection during processing and storage typically relies on dense internal networks, stable interfacial structures, and reduced molecular diffusivity [39]. However, these same structural characteristics may inherently impede digestive disassembly and subsequent nutrient release. Consequently, structural stability and release efficiency represent a pair of fundamentally antagonistic design parameters in food-grade delivery systems.

From a design-oriented perspective, the kinetics of release can be just as important as encapsulation itself in sports nutrition applications. Unlike conventional fortified foods, sports nutrition products are often consumed during specific physiological windows related to exercise, recovery, and increased metabolic demand [12]. Therefore, delivery systems must not only ensure the stability of bioactive compounds during processing and storage but also time the nutrient release to match gastrointestinal absorption dynamics and the physiological needs following exercise. This is particularly critical for bioactives that require rapid uptake in the intestine, such as amino acids, antioxidants, and functional compounds related to exercise.

Several structural parameters, including network compactness, interfacial organization, and matrix digestibility, play significant roles in influencing release behavior during gastrointestinal transit. Delivery structures that are too compact or highly cross-linked may show excellent stability during storage but can restrict enzyme accessibility and delay nutrient release during digestion [40,41]. Conversely, structures that are too loose may disassemble prematurely during storage or in the gastric phase, leading to early leakage, degradation, or reduced functional efficacy [42,43]. Therefore, effective delivery system design requires a balance between structural robustness and digestive responsiveness, rather than simply maximizing either protection or release.

To tackle this challenge, food-grade delivery systems increasingly prioritize “stage-specific functionality.” In this approach, delivery structures play distinct roles at different stages of their lifecycle. During food processing and storage, delivery systems primarily act as protective structures that maintain the physical and chemical stability of bioactive compounds [44]. In contrast, during gastrointestinal digestion—especially in the small intestine—these delivery structures are expected to undergo enzymatic degradation or restructuring induced by bile salts, thereby facilitating the release and absorption of encapsulated bioactives [45]. Importantly, the objective of food-grade delivery systems is not prolonged retention or maximal sustained release, but rather a temporally coordinated release that balances gastric tolerance with efficient nutrient availability in the intestine [44]. This digestion-centered, stage-specific functionality is increasingly recognized as a key principle that distinguishes food delivery systems from pharmaceutical delivery systems.

### 2.3. Food-Grade and Sports Nutrition–Specific Design Constraints

Food-grade delivery systems, especially in sports nutrition applications, face more stringent and complex constraints compared to pharmaceutical delivery systems. This distinction is crucial because sports nutrition products are often consumed during physically demanding activities. Firstly, all materials used in these delivery systems must comply with food safety regulations and demonstrate adequate gastrointestinal tolerance [46]. Since sports nutrition products are usually consumed in quick succession and may contain high levels of active ingredients, it is essential for the delivery materials to maintain safety and tolerability during frequent use [47]. Therefore, effective food-grade delivery systems must be designed not only for functional performance but also for long-term safety and compatibility with the digestive system under realistic consumption conditions.

In addition to safety concerns, sensory compatibility is a crucial design constraint in sports nutrition delivery systems. Most sports nutrition products are offered as beverages, gels, or ready-to-consume supplements, where rapid ingestion, ease of swallowing, and acceptable mouthfeel are essential [48,49]. Delivery structures that significantly increase viscosity, create noticeable particulate sensations, or disrupt flow behavior can negatively impact product acceptability during exercise or recovery [50]. Furthermore, gastrointestinal tolerance is closely tied to these sensory and structural features. During exercise and recovery, the gastrointestinal system experiences altered blood flow, faster gastric emptying, and heightened physiological stress. In such situations, delivery systems that excessively delay gastric emptying, raise osmotic burden, or form hard-to-digest aggregates may adversely affect gastrointestinal comfort and nutrient absorption [51]. Consequently, food-grade delivery systems in sports nutrition are increasingly expected to function in a relatively “invisible” manner, providing structural and nutritional benefits without significantly compromising sensory perception or physiological tolerance [52].

Lastly, the ability to process these systems industrially and their cost-effectiveness are critical factors in translating them into commercial sports nutrition products. Unlike laboratory-scale systems, industrial food production relies heavily on established manufacturing processes such as mixing, homogenization, thermal treatment, extrusion, and shaping [53]. Delivery structures that require overly complex fabrication methods, specialized equipment, or costly ingredients are unlikely to be implemented at a large scale. Moreover, the structural functionality of these systems must remain robust under typical manufacturing conditions, as processing can lead to rearrangements, aggregation, or phase instability, directly affecting release behavior and nutritional performance. Consequently, delivery systems that can be formed, processed, and maintained under conventional food manufacturing conditions are generally more compatible with the scalability, consistency, and cost-effectiveness needed for sports nutrition applications.

## 3. Emerging Gel-Based Delivery Architectures and Responsive Design Strategies

After clarifying the core design principles of food-grade delivery systems, a key question naturally follows: through what structural formats and construction strategies can these principles be implemented in real food matrices? Unlike traditional reviews that mainly categorize systems by the type of material used, recent evidence indicates that delivery performance is not solely dependent on the source of the ingredients. Instead, it is fundamentally influenced by the spatial architecture, the synergy between components, and their dynamic responses during digestion [54]. This is particularly important in sports nutrition applications, where delivery structures must carefully balance processing strength, sensory compatibility, and the efficiency of digestion-enabled release. In this context, the combination of “structure and strategy” serves as a crucial link between design concepts and their practical application.

### 3.1. Protein Gel-Based Self-Assembled Delivery Structures

**Figure 2:**
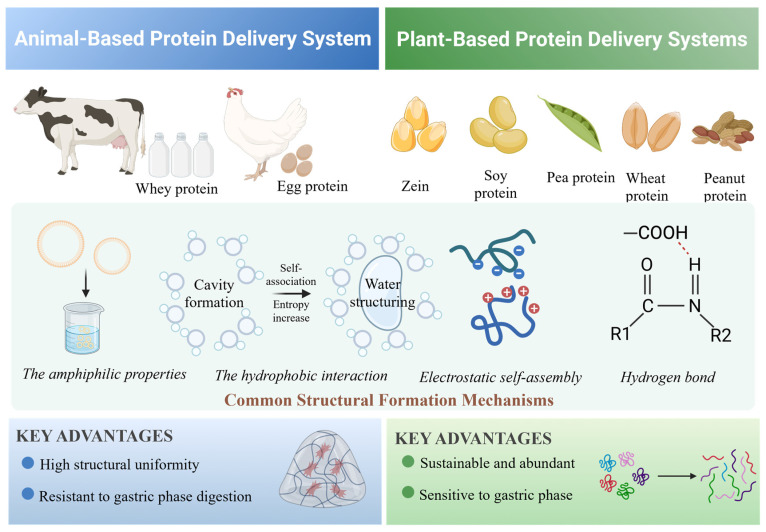
Protein gel-based self-assembled delivery systems derived from animal and plant proteins. Animal-derived proteins, such as whey protein and egg white protein, and plant-derived proteins, including zein, soy protein, and pea protein, can form diverse delivery architectures including nanoparticles, microgels, nanogels, and protein-stabilized emulsions.

Proteins are recognized as some of the most promising building blocks for food delivery systems due to their safety for consumption, compatibility with physiological processes, and ability to be structurally modified. Proteins from various sources exhibit significant differences in their molecular structure, interfacial behavior, and digestive properties, all of which directly influence their assembly processes and functional effectiveness in delivery applications [55]. As illustrated in Figure 2, protein-based delivery systems are generally categorized into two groups: animal-derived proteins and plant-derived proteins. Despite their differing origins, both categories share several common assembly principles. These include self-assembly driven by amphiphilicity, hydrophobic interactions, electrostatic attractions, hydrogen bonding, and water-mediated structural organization. Together, these intermolecular interactions determine the formation of nano- and micro-scale gel-like delivery structures, which ultimately affect encapsulation, structural stability, and digestive responsiveness in functional food systems.

#### 3.1.1. Animal-Derived Protein Gel-Based Delivery Systems

Animal-derived proteins, particularly those from dairy and egg whites, are a well-researched and practical category of food-grade delivery materials [56]. Their advantages are generally linked to high structural uniformity, well-defined functional properties, and strong adaptability to food processing operations.

Whey protein is one of the most extensively studied examples of animal-derived delivery systems. Its key components include β-lactoglobulin, α-lactalbumin, and bovine serum albumin [57]. These globular proteins have distinct tertiary structures and hydrophobic binding sites, which allow them to form relatively stable complexes with various hydrophobic bioactives through hydrophobic interactions, hydrogen bonding, and electrostatic forces [58]. The compact tertiary structures and amphiphilic nature of whey proteins facilitate controlled self-assembly during heat treatment or interfacial rearrangement, resulting in the formation of structurally uniform nanogels, protein aggregates, and emulsion-based carriers [59]. This structural uniformity is crucial for improving the consistency of encapsulation and controlling the release of contents triggered by digestion. Research has demonstrated that whey proteins can be engineered into nanoparticles, nanogels, or protein-stabilized emulsions using methods such as heat induction, self-assembly, or interfacial adsorption [60,61,62]. These structures effectively protect encapsulated bioactive compounds during processing and storage while enabling progressive release during digestion.

Notably, whey proteins exhibit a degree of resistance to pepsin in the stomach. Specifically, β-lactoglobulin can maintain partial conformational integrity under acidic conditions, which helps delay premature release in the gastric phase. As disassembly occurs under the combined effects of pancreatic proteases and bile salts in the small intestine, bioavailability can be enhanced [63]. This “gastric protection–intestinal release” behavior offers a significant functional advantage in sports nutrition, particularly for bioactives that need rapid intestinal uptake while avoiding early gastric inactivation [64].

In addition to whey proteins, egg white proteins are also commonly used for developing food delivery systems. Egg white proteins, with ovalbumin as the primary component, display excellent film-forming and gelation capabilities, allowing them to create continuous network structures under thermal treatment or pH modulation [65]. Research has noted that egg-white-protein-based systems can encapsulate polyphenols, vitamins, and lipophilic bioactives by forming microgels or composite networks. These systems often exhibit gradual structural breakdown during digestion, which promotes release in the intestinal stage [66,67].

Overall, animal-derived protein delivery systems offer significant advantages in terms of structural controllability and functional stability, making their applications in sports nutrition relatively advanced. However, reliance on animal-derived sources and acceptance issues among certain populations have sparked growing interest in plant-protein-based systems.

#### 3.1.2. Plant-Derived Protein Gel-Based Delivery Systems

The rapid growth of plant-based diets and the increasing demand for sustainable foods have led to heightened interest in plant-derived proteins within food delivery research. Compared to animal-derived proteins, plant proteins are more abundant and typically have a lower environmental impact. However, their complexity and functional variability present challenges in designing effective delivery systems [68].

Plant proteins are often sourced from cereals, legumes, oilseeds, and pseudocereals, each having diverse molecular structures and variations in hydrophobicity and conformational flexibility [69]. Studies have shown that proteins such as zein, soy protein, pea protein, and wheat gliadin can form microparticles, nanoparticles, or protein-stabilized emulsions through self-assembly and interfacial adsorption, which aids in the delivery of lipophilic bioactive compounds [70,71].

In comparison to animal proteins, plant proteins usually have limited solubility, reduced native structural stability, and lower interfacial activity. As a result, many studies focus on improving delivery performance using physical treatments, enzymatic methods, or by complexing with other food-grade components like polysaccharides and lipids [69,72]. Among these strategies, plant protein–polysaccharide complexes are notably effective, as they can enhance structural stability and responsiveness without greatly compromising food-grade qualities [73].

From a digestion standpoint, plant-protein-based delivery systems are often more sensitive to pepsin, which can lead to partial structural breakdown occurring as early as the gastric phase [74]. This characteristic can be advantageous for sports nutrition, as it may facilitate quicker release for post-exercise recovery products, provided that appropriate design ensures processing and storage stability.

In summary, plant protein delivery systems offer significant benefits in terms of sustainability and consumer appeal. However, challenges remain regarding structural control and consistency in performance. Consequently, current research is increasingly adopting a design-driven approach to maximize the delivery potential of plant proteins through structural regulation and composite strategies.

#### 3.1.3. Comparative Evaluation and Design Considerations of Protein-Based Delivery Systems

From a design-oriented perspective, comparing animal-derived and plant-derived protein delivery systems should not be limited to a simple evaluation of encapsulation efficiency or structural stability. Instead, their suitability depends on the nutritional objectives and delivery requirements of the target application. Animal-derived proteins generally exhibit more predictable self-assembly behavior, stronger interfacial stabilization, and greater resistance to premature disassembly in the gastric environment. This makes them particularly effective at protecting oxidation-sensitive, poorly soluble, or easily damaged bioactive compounds during storage and gastric transit. For example, whey protein isolate nanofibrils have been shown to self-assemble on the surface of *Lactiplantibacillus plantarum*, improving probiotic protection against environmental stress and 3D-printing processing; the resulting composite gels maintained viable cell counts above 8.0 log CFU/g and showed stable 3D-printed structures, indicating the value of whey-derived gel architectures for protecting sensitive functional ingredients under processing stress [75]. Another example is the combination of heat-treated whey protein isolate with sodium alginate, which forms soluble self-assembled biopolymer complexes. In this case, the deposition of polysaccharides on whey protein aggregates was proposed to protect hydrophobic ligands associated with protein hydrophobic sites, showcasing the delivery potential of animal protein–polysaccharide assemblies for lipophilic bioactives [76].

In contrast, plant-derived protein systems often exhibit greater digestive responsiveness and earlier structural breakdown under gastrointestinal conditions. While this may diminish storage robustness, it can be beneficial in post-exercise nutrition scenarios where rapid nutrient availability is essential. For instance, corn fiber gum and soybean protein isolate double-network hydrogels have been developed at room temperature for riboflavin delivery. The 0.25% corn fiber gum formulation showed low swelling in simulated gastric fluid while swelling more in simulated intestinal fluid, with intestinal release driven by swelling, matrix erosion, and proteolysis of the soybean protein isolate [77]. This example illustrates how plant protein–polysaccharide hydrogels can be designed for gastric protection followed by intestinal release. Similarly, soy protein isolate/κ-carrageenan-stabilized Pickering high internal phase emulsions improved the stability, digestion stability, intestinal bioaccessibility, cellular uptake, and bioactivity of nobiletin, suggesting that plant protein–polysaccharide interfacial systems may be especially useful for hydrophobic bioactives with poor solubility and pH sensitivity [78].

However, these examples also highlight that neither animal- nor plant-derived protein systems are universally superior. Whey-based systems show clear advantages in structural uniformity and protection, but their performance may depend strongly on controlled heat treatment, interfacial assembly, shear history, and formulation composition, which can complicate large-scale manufacturing [79]. On the other hand, plant protein systems offer sustainability and stronger compatibility with plant-based sports nutrition products, but they often require polysaccharide or lipid-assisted structuring to overcome limitations in solubility, interfacial activity, and reproducibility [80]. Therefore, the selection of protein-based gel delivery systems should be guided not solely by encapsulation efficiency, but by a comprehensive assessment of processing robustness, storage stability, digestive release behavior, scalability, target bioactive properties, and the specific sports nutrition context.

### 3.2. Multicomponent Hybrid Delivery Systems

Protein-based self-assembled systems provide significant advantages in terms of food-grade safety and structural adaptability. However, delivery systems constructed from a single component often face challenges in achieving multiple design goals, including stability, protection efficiency, and controlled release. To address these challenges, researchers are increasingly focusing on developing multicomponent hybrid delivery systems in the area of food delivery [81]. By integrating additional food-grade components, like polysaccharides or lipids, it is possible to enhance interfacial properties, mechanical stability, and digestive behavior synergistically, all while keeping processing complexity relatively low [82].

#### 3.2.1. Protein–Polysaccharide Hybrid Delivery Systems

Protein–polysaccharide systems are among the most extensively studied types of multicomponent delivery structures. These systems are typically formed through electrostatic interactions, hydrogen bonding, or hydrophobic associations, leading to the creation of composite interfaces or three-dimensional networks [83]. Depending on the processing conditions and the composition of the components, these interactions can drive the formation of various structural organizations. These include linear complexes, pearl-chain assemblies, knot-like aggregates, tape-like structures, spherical particles, and continuous network architectures. This structural diversity offers significant flexibility in regulating interfacial behavior, rheological properties, and digestive responsiveness in food-grade delivery systems [83]. Studies have shown that incorporating polysaccharides can notably enhance the stability of protein-based systems in complex food environments. This is particularly important near the protein isoelectric point or in conditions with high ionic strength, where composite layers or coating structures can help reduce risks of protein aggregation and sedimentation [84,85].

In emulsion-based delivery systems, protein–polysaccharide composite interfaces are commonly utilized to influence lipid digestion. Multilayer interfaces or crosslinked composite layers can modify interfacial thickness and permeability, as well as enzyme accessibility. This alteration in structure slows lipid hydrolysis and regulates the release behavior of lipophilic bioactives [86]. This approach—connecting interfacial structure design to digestion kinetics—has been recognized as a key feature that differentiates food delivery systems from pharmaceutical delivery systems [87].

Additionally, protein–polysaccharide complexes are often employed to create microgels or filled hydrogel structures, in which lipid droplets or hydrophobic bioactives are embedded within protein–polysaccharide networks [88]. These structures can demonstrate high physical stability during processing and storage, while also undergoing pH-driven or enzyme-mediated changes in the gastrointestinal environment. This mechanism facilitates the targeted release of bioactives during digestion [89].

#### 3.2.2. Protein–Lipid Hybrid Delivery Systems

Protein–lipid hybrid systems are primarily designed to deliver hydrophobic bioactive compounds. The core structural formats of these systems include protein-stabilized emulsions, nanoemulsions, and filled or composite colloidal particles [90]. In these systems, the lipid phase acts as the main carrier for lipophilic compounds, while proteins provide stability and adjustable functionality through interfacial adsorption or self-assembly [91]. Typically, these hybrid structures are created using high-speed homogenization, high-pressure homogenization, or interfacial self-assembly processes. These methods help regulate droplet size distribution, interfacial compactness, and colloidal stability.

Evidence suggests that protein-stabilized oil-in-water emulsions not only enhance the dispersibility of hydrophobic bioactives in aqueous food products but they also impact lipid digestion rates and bioaccessibility. This is achieved by regulating the physical state of the lipid phase—whether it is liquid or partially crystalline—and by altering the architecture at the interface [92]. Generally, higher lipid crystallinity reduces enzymatic accessibility, which indirectly influences the release rate of bioactives [93]. During gastrointestinal digestion, bile salt adsorption and enzymatic hydrolysis can progressively remodel protein–lipid interfaces. This remodeling alters interfacial permeability and accelerates structural disassembly, thus influencing the kinetics of lipid digestion and the intestinal bioaccessibility of the encapsulated compounds [94].

More complex designs can further integrate protein–lipid systems with polysaccharides to create multilayer interfaces or filled hydrogel structures [95]. These multicomponent architectures can “physically confine” lipid droplets within protein or protein–polysaccharide networks, allowing for coordinated control over structural stability and digestive behavior. This approach offers greater design flexibility to balance processing stability with digestion-enabled release [80].

Importantly, these multicomponent hybrid systems are not just a simple additive combination of material functions. Instead, they achieve refined regulation of delivery behavior by orchestrating the spatial distribution of each component and their interactions [96]. Compared to single-component systems, hybrid structures offer greater flexibility in tuning interfacial properties, digestion kinetics, and release pathways—traits that are particularly beneficial for sports nutrition applications, which often require a rapid transition from “protection” to “release” within a limited time frame [97].

**Figure 3:**
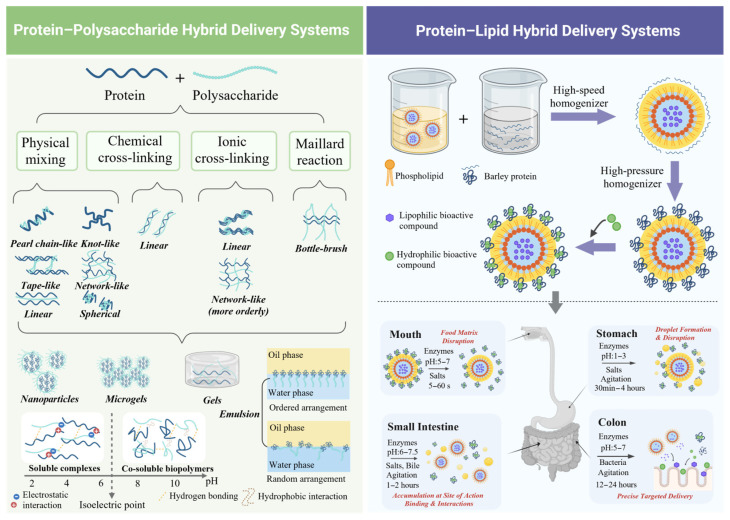
Multicomponent hybrid gel-based delivery systems constructed through protein–polysaccharide and protein–lipid interactions for functional food applications. Protein–polysaccharide systems can form diverse delivery architectures, including soluble complexes, nanoparticles, microgels, hydrogels, and emulsion-based structures. Protein–lipid hybrid systems mainly include protein-stabilized emulsions, nanoemulsions, and composite colloidal structures for encapsulation of lipophilic bioactives.

Overall, multicomponent hybrid delivery systems offer food-grade architectures that integrate stability, functionality, and design flexibility. They also establish a structural foundation for the development of environmentally responsive delivery systems. To illustrate this concept further, a multi-component delivery system with potential applications in sports nutrition is depicted in Figure 3.

### 3.3. Responsive and Digestion-Triggered Gel Delivery Systems

Sports nutrition products are usually consumed during exercise or soon after, when the gastrointestinal tract is in a highly dynamic physiological state. This state is marked by accelerated gastric emptying, increased secretion of digestive enzymes, and changes at lipid and protein interfaces mediated by bile salts [12]. Given these conditions, delivery systems must not only remain stable during processing and storage but must also respond to crucial physiological signals during digestion. This responsiveness ensures that the structure transforms and nutrients are released at the appropriate times [98]. In sports nutrition, timing is vital, as nutrient availability correlates closely with exercise intensity, recovery needs, and gastrointestinal tolerance.

Mechanistically, food-grade responsive delivery systems do not rely on complex external stimuli. Instead, they primarily utilize intrinsic physicochemical and enzymatic variations along the gastrointestinal tract, particularly pH gradients, digestive proteases, and bile salt-mediated changes. These factors influence the stability and breakdown behavior of protein-based delivery structures during digestion [99,100]. From a design perspective, the shifts in the digestive environment present opportunities to develop delivery systems that respond at specific stages and release nutrients in a timely manner.

During the gastric phase, low pH can significantly affect protein structure and intermolecular interactions [101]. Under acidic conditions, surface charges may become partially neutralized, reducing electrostatic repulsion and enhancing hydrophobic associations and intermolecular aggregation. Consequently, protein-based delivery structures may experience temporary densification or aggregation [65,102], which can help limit early nutrient release and improve tolerance within the stomach. Although pepsin can act on the protein backbone, its disruption of the overall delivery architecture is usually limited during short gastric residence times, allowing protein-based carriers to largely remain intact in this phase [103]. In sports nutrition, maintaining this temporary structure may help minimize gastrointestinal discomfort associated with exercise and prevent excessive osmotic loading during gastric transit.

Once the contents reach the small intestine, the digestive environment changes considerably. The pH becomes neutral or weakly alkaline, proteins regain a net charge, and intermolecular repulsion increases. Concurrently, pancreatic enzymes greatly enhance the breakdown of the protein scaffold, loosening or fragmenting protein networks [104]. Additionally, bile salts act as natural surfactants that can penetrate protein–lipid or protein–water interfaces, altering interfacial tension and molecular packing. These changes promote the disassembly or restructuring of delivery structures [94], increasing the structural openness during the intestinal phase and facilitating the release and absorption of encapsulated nutrients.

It is important to understand that this digestion-triggered structural evolution should not be seen as mere “passive destabilization” but rather as a functional feature that can be leveraged. By adjusting the type of protein, the compactness of the structure, and interactions with other food-grade components, the stability of the delivery structure in the stomach and its disassembly rate in the small intestine can be fine-tuned within a controllable range. This allows for regulation of the nutrient release timeline [21,32].

The practical importance of digestion-triggered delivery systems becomes especially apparent in exercise-related gastrointestinal conditions, where nutrient timing, gastrointestinal tolerance, and absorption efficiency are closely linked to the stage of exercise and physiological demands. During prolonged or high-intensity exercise, gastrointestinal physiology can experience significant changes, such as the redistribution of splanchnic blood flow, faster gastric emptying, increased osmotic sensitivity, and a higher likelihood of gastrointestinal discomfort [105]. Under these circumstances, delivery systems optimized solely for storage stability may exhibit inadequate nutrient release or poor gastrointestinal tolerance. Therefore, sports nutrition delivery systems increasingly need to feature structurally responsive behaviors that can adapt to the dynamically changing digestive conditions before, during, and after exercise [51].

Before exercise, the primary goal of responsive delivery systems is to ensure sustained availability of nutrients while minimizing rapid fluctuations in blood sugar levels and gastrointestinal stress. Low-glycemic carbohydrate gels made with alginate, pectin, or modified starch matrices can slow diffusion rates and gastric emptying of free sugars, supporting a more gradual release of glucose [106]. Composite gel systems that combine both fast- and slow-release carbohydrate fractions may help to moderate glycemic response profiles and reduce sudden insulin spikes [107]. In addition, weakly crosslinked structures that respond to pH or hydration changes may enhance swelling and nutrient diffusion in the intestines, thereby improving absorption efficiency without overly burdening the stomach [108]. Sensory and rheological properties, such as viscosity, sweetness persistence, and adhesiveness, are also crucial for maintaining gastrointestinal comfort and user compliance before exercise.

During exercise, responsive delivery systems need to meet several requirements simultaneously: portability, rapid nutrient availability, gastrointestinal tolerance, and osmotic control. Compared to sports drinks and solid nutrition bars, gel-based systems can provide a higher effective carbohydrate density while remaining easy to consume. Their structured networks may buffer against gastric shear stress and mechanical agitation caused by vigorous movement, reducing the risks of reflux, cramping, and bloating during endurance activities [51]. For example, ionically crosslinked alginate–pectin gels can form soft structures in the stomach that quickly break down in the intestines, leading to smoother carbohydrate release profiles and improved gastrointestinal tolerance [109]. Delivery systems that incorporate multiple carbohydrate sources—such as glucose and fructose, which utilize different intestinal transporters—may further enhance carbohydrate oxidation efficiency during prolonged exercise [47]. It is essential to modulate gel strength, hydration capacity, and osmotic load appropriately; excessive viscosity or hyperosmotic formulations can hinder gastric emptying and nutrient absorption during exercise. In endurance sports like marathon running, cycling, and triathlons, responsive gel systems may also include electrolytes such as sodium, potassium, and magnesium to assist with fluid balance and electrolyte replacement under varying environmental conditions [110].

After exercise, digestion-triggered delivery systems are particularly valuable for facilitating glycogen recovery, muscle protein synthesis, and reducing oxidative stress. Protein-based gels and composite matrices can prolong amino acid availability by slowing down their digestive breakdown, thus supporting a sustained supply of substrates for muscle repair [111]. When combined with carbohydrates, these systems can achieve staged nutrient delivery—rapidly available carbohydrates support initial glycogen replenishment while the slower matrix disassembly maintains nutrient availability during recovery [112]. Responsive protein–polysaccharide composite systems also show strong potential for encapsulating oxidation-sensitive bioactive compounds like curcumin, quercetin, carotenoids, polyphenols, and various vitamins [113]. In such cases, digestion-triggered restructuring may significantly improve intestinal bioaccessibility during the post-exercise recovery phase. Probiotics and prebiotics incorporated into gel matrices may be protected during gastric transit and released in the distal intestine, contributing to inflammation reduction and epithelial recovery after exercise-related physiological stress [44].

Moreover, digestion-responsive matrices offer strategic advantages for delivering amino acids and ergogenic compounds that are closely linked to sports performance and recovery, such as BCAAs, creatine, glutamine, and β-alanine. Instead of causing excessively rapid spikes in plasma concentrations, structured gel and hydrogel matrices can facilitate more physiologically coordinated nutrient availability by synchronizing the release from the intestines with the metabolic demands and absorption kinetics following exercise. From a design perspective, responsiveness in sports nutrition systems is often achieved by adjusting the matrix’s compactness, crosslinking density, hydration behavior, and interfacial structure [114]. For instance, weakly crosslinked hydrogel networks can enhance intestinal swelling and nutrient diffusion, while multilayer protein–polysaccharide interfaces can delay premature gastric release and improve oxidative stability during storage [83].

Overall, responsive and digestion-triggered delivery systems represent a food-grade approach that aligns structural changes with the dynamic physiology of the gastrointestinal system during exercise. Rather than focusing on structural permanence, these systems are increasingly engineered to achieve well-timed transitions between gastric protection, intestinal release, and post-exercise nutrient utilization. Such exercise-stage-responsive systems offer a promising solution for optimizing nutritional support throughout the various phases of physical activity.

### 3.4. Exercise-Specific Gastrointestinal Physiology and Its Implications for Delivery System Design

Exercise-specific gastrointestinal physiology imposes constraints on delivery system design that are not fully captured by conventional static digestion models. During prolonged or high-intensity exercise, blood flow is redirected from the splanchnic region (which supplies the intestines) to active skeletal muscles. This reduced intestinal perfusion can slow down epithelial transport, change nutrient absorption rates, and increase the likelihood of gastrointestinal discomfort [115]. In these circumstances, delivery systems that rapidly release large amounts of free nutrients in the stomach or proximal intestine may lead to high local osmotic pressure. This can cause fluid shifts into the intestinal lumen and raise the risk of bloating, cramping, nausea, or diarrhea [116]. Therefore, delivery systems for use during exercise should not be designed solely to maximize release rate; rather, they should coordinate release with the reduced absorptive capacity and altered motility of the exercising gut.

From this standpoint, it is essential to explicitly consider the relationship between delivery structure and gastrointestinal distress. Highly concentrated carbohydrate solutions, rapidly dissolving matrices, and hyperosmotic formulations may increase osmotic load and delay gastric emptying, thereby aggravating gastrointestinal symptoms during exercise [117]. Similarly, delivery structures with excessive viscosity or strong gel strength may slow gastric transit and create a sensation of fullness. In contrast, poorly dispersed emulsions or unstable colloidal systems may undergo coalescence, phase separation, or delayed lipid digestion, further increasing gastrointestinal burden [118]. On the other hand, moderately structured gels, weakly crosslinked hydrogels, and ionically responsive alginate–pectin systems can reduce discomfort by controlling nutrient diffusion, buffering gastric shear, and preventing sudden osmotic spikes, all while allowing for intestinal disassembly and nutrient release [119].

These physiological constraints translate into several practical design requirements. First, delivery systems intended for intake during exercise should maintain low-to-moderate viscosity and avoid excessive gel rigidity to preserve swallowability and gastric emptying. Second, gels loaded with carbohydrates or amino acids should regulate diffusion to prevent abrupt nutrient release, especially when intestinal blood flow and absorptive capacity are compromised [120]. Third, hydrophobic bioactives delivered through emulsions or emulsion gels should remain physically stable during storage and gastric transit but should avoid excessive lipid loading that may slow gastric emptying [121]. Fourth, systems designed for post-exercise recovery can afford to have stronger structural matrices than those needed during exercise since gastrointestinal perfusion and digestive capacity gradually improve after exercise.

**Figure 4:**
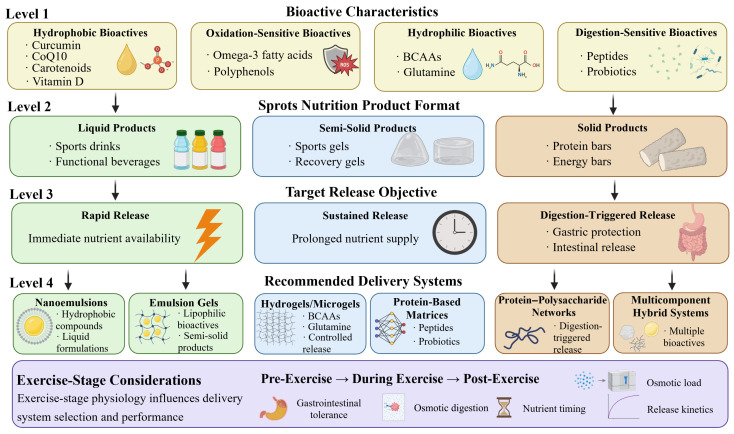
Design-driven framework for selecting food-grade delivery systems in sports nutrition applications. The framework illustrates how “structure and strategy” can be integrated to match bioactive characteristics, product format, target release profile, and exercise-stage requirements with appropriate delivery architectures. Different delivery systems, including nanoemulsions, emulsion gels, hydrogels, protein-based matrices, protein–polysaccharide networks, and multicomponent hybrid systems, provide distinct advantages depending on the intended application. Exercise-associated physiological factors, including gastrointestinal tolerance, osmotic load, nutrient timing, and release kinetics, should be considered during delivery system selection.

In summary, the selection of delivery systems in sports nutrition should not be based solely on encapsulation efficiency, structural stability, or release rate. Instead, “structure and strategy” should be understood as the coordinated matching of bioactive properties, product format, target release profile, and exercise-stage requirements with appropriate delivery architectures. For example, hydrophobic or oxidation-sensitive bioactives are generally better suited to nanoemulsions, emulsion gels, or multilayer interfacial systems, whereas hydrophilic compounds, peptides, probiotics, and digestion-sensitive ingredients may require hydrogels, microgels, protein matrices, or protein–polysaccharide networks. Liquid products favor small, well-dispersed, low-viscosity systems, while semi-solid gels and solid matrices can accommodate more complex structures for digestion-regulated release. Based on this rationale, a design decision framework for matching bioactive types, product formats, release objectives, and delivery architectures is illustrated in Figure 4. Remaining scientific and engineering challenges are discussed in the following section.

## 4. Application of Food Delivery Systems in Real Sports Nutrition Products

Unlike model delivery systems developed in simplified laboratory settings, real world sports nutrition products encounter additional formulation challenges. These challenges are related to processing, sensory perception, gastrointestinal tolerance, and specific consumption scenarios. As a result, the practical effectiveness of food delivery systems in sports nutrition relies not only on their structural design but also on their compatibility with realistic food matrices and ingestion behaviors. Different classes of bioactive compounds have unique physicochemical properties and stability challenges, which often necessitate different delivery strategies based on the target product format. To enable a clear comparison of current delivery approaches, Table 1 summarizes representative food-grade delivery systems according to bioactive type, structural characteristics, processing stability, release behavior, and potential applications in sports nutrition.

### 4.1. Liquid Sports Nutrition Products

Liquid products are among the most common formats used in sports nutrition. Research indicates that athletes involved in endurance and high-intensity training often replenish carbohydrates through sports drinks in liquid form [134]. When incorporating bioactive compounds into sports nutrition systems, it is essential to consider factors like commercial viability and real-world benefits. This consideration often depends on the ability of these bioactives to be stably dispersed in a liquid matrix while preserving acceptable flavor, gastrointestinal tolerance, and effective release during digestion [135].

From a commercial perspective, developing liquid sports nutrition products involves more than just carbohydrate replenishment. It requires balancing hydration efficiency, electrolyte replacement, gastrointestinal tolerance, osmolality control, and ensuring compatibility with functional ingredients and delivery systems. As summarized in Table 2, typically, isotonic sports drinks utilize carbohydrates such as glucose, fructose, sucrose, and maltodextrin (a type of glucose polymer) as their main sources. These carbohydrates are often paired with electrolytes, such as sodium [136]. In recent years, glucose polymers have gained popularity because they can deliver fairly high carbohydrate levels without significantly raising osmotic pressure [137]. Generally, sports drinks are formulated with a carbohydrate concentration of about 6 to 8%, which is considered optimal for promoting rapid gastric emptying and efficient intestinal fluid absorption [138]. Exceeding this range may lead to gastrointestinal discomfort and negatively impact nutrient delivery efficiency.

Independent evaluations of commercially available maltodextrin/fructose (MD+F) sports drinks have revealed a clear chain of evidence regarding their effectiveness. One study compared a commercial MD+F formulation with an isocaloric single-carbohydrate maltodextrin drink and a placebo. The findings indicated that mixed-carbohydrate strategies led to higher exogenous carbohydrate oxidation and improved performance outcomes under the tested conditions [162]. However, the authors pointed out that high carbohydrate intake in real-life situations may be limited by tolerability and gastrointestinal issues. Excessively high intake may not be feasible for everyone, which sets practical upper limits on product formulation.

Traditionally, sports drinks have focused on hydration, carbohydrate supply, and electrolyte replacement. However, there is an increasing trend in sports nutrition towards “recovery and functional enhancement.” For example, Tirla et al. [163] observed a shift toward the incorporation of functional ingredients such as BCAAs, glutamine, electrolytes, and vitamins in gel-type products. A similar trend is occurring with liquid products, but with stricter constraints. Many potential functional components, particularly those that are hydrophobic or prone to oxidation, have poor dispersibility in aqueous solutions, limited storage stability, and pose challenges in flavor masking. Furthermore, these components are likely to undergo sedimentation, aggregation, or loss of activity during processing and storage [11].

To address these issues, the introduction of delivery systems in liquid sports nutrition products aims to enhance encapsulation efficiency while achieving three specific objectives: (i) improve the dispersibility and physical stability of functional ingredients without compromising low viscosity and taste; (ii) minimize the risks of aggregation, sedimentation, and chemical degradation over the product’s shelf life; and (iii) ensure that the ingredients are bioaccessible during digestion without increasing gastrointestinal discomfort. This approach helps to prevent situations where a product remains stable during storage but fails to release active ingredients during digestion.

### 4.2. Solid and Semi-Solid Sports Nutrition Products

Compared to liquid sports nutrition products, solid and semi-solid foods—such as energy bars, protein bars, sports gels, and soft solid supplements—differ significantly in how they are consumed, their gastrointestinal kinetics, and their digestion behavior. These products typically have higher energy density and stronger structural integrity. The release of nutrients is influenced not only by the formulation composition but also by the macro- and micro-structural characteristics of the food matrix [164]. In solid and semi-solid sports nutrition, delivery systems are not just microscopic carriers; they are intimately connected with the food structure and can directly impact digestion processes and the timing of nutrient release [165].

Energy bars and protein bars are prime examples of solid sports nutrition products. They generally consist of proteins, carbohydrates, lipids, and binders, which create multiphase solid structures with specific mechanical strength [166]. In these products, bioactive compounds are often not present as free molecules. Instead, they are incorporated within protein networks, lipid domains, or composite matrices, and their release is highly dependent on how the food is broken down during oral mastication and subsequent disintegration in the stomach [167].

In practical applications, the main design priority is often not rapid release but rather the prevention of migration, aggregation, or degradation of bioactives during processing and storage. Additionally, it is important to modulate the release rates during digestion through structural restrictions [168]. Protein-based structures commonly serve dual functions in these products: they provide essential nutrients while also acting as structural scaffolds that physically embed functional compounds within the food matrix [57]. The particle size distribution created during mastication, combined with the stepwise breakdown of the matrix by stomach mechanical forces and chemical digestion, determines the release pathway and the relevant time scale for bioactive delivery [45,169].

Unlike liquid products, bar-type products allow the delivery systems to be present in a “perceptible but not intrusive” manner. The structural integrity of the matrix itself can be utilized to delay nutrient release [170]. However, this requires careful design to avoid overly dense or digestion-resistant structures, which may hinder the release of bioactives in the gastrointestinal tract and consequently reduce overall utilization efficiency.

Sports gels and other semi-solid supplement foods have a unique role in sports nutrition. These products often exhibit shear-thinning or pseudoplastic rheological behavior, which makes them easier to swallow while still maintaining some structural integrity after ingestion [171]. Compared with liquid drinks, their digestion is more strongly governed by structural disintegration rather than simple dissolution.

In these products, delivery structures commonly exist as networks, microgels, or composite architectures. The release of bioactive compounds depends on the gradual loosening and restructuring of these structures under gastrointestinal conditions [172]. During the gastric phase, semi-solid structures may retain some integrity, which limits premature release and reduces the risk of gastric irritation [173]. When these structures enter the small intestine, enzymatic action and bile-salt-mediated rearrangements promote the disassembly of the delivery system, facilitating the release and absorption of bioactives [174].

This mechanism of structure evolution-driven release can provide advantages for “stage-specific replenishment” in sports nutrition. However, the design window is relatively narrow: overly loose structures may compromise storage stability and oral experience, while overly rigid structures may delay digestive breakdown and reduce replenishment efficiency [175]. Thus, designing delivery systems in semi-solid formats requires a careful balance between structural stability, gastrointestinal tolerability, and release efficiency.

Overall, in solid and semi-solid sports nutrition, the functional role of delivery systems goes beyond simply improving dispersibility or stability of bioactives. By co-structuring with the food matrix, delivery systems can play a role in regulating digestion kinetics and release behavior as a whole. While these systems allow for greater design flexibility, they also increase the risk of structural failure impacting sensory perception and physiological tolerability. Consequently, the design of delivery systems for solid and semi-solid sports nutrition should prioritize compatibility with the overall food structure and must be evaluated in realistic contexts of chewing and digestion. Only when structural functionality aligns with actual eating behavior can these delivery systems provide significant value in solid and semi-solid sports nutrition products (Table 3).

### 4.3. Comparative Evaluation of Delivery Strategies for Sports Nutrition Applications

The preceding sections illustrate that food-grade delivery systems can be effectively integrated into a wide range of sports nutrition products, including beverages, protein bars, recovery supplements, and sports gels. However, directly comparing different delivery strategies can be challenging. This is because each system is typically designed to address unique formulation constraints, bioactive characteristics, and physiological goals. As a result, no single delivery platform can be considered universally optimal for all sports nutrition applications.

From the perspective of bioactive properties, different delivery systems exhibit distinct functional advantages. Emulsion-based and nanoemulsion systems are generally more suitable for hydrophobic compounds, such as curcumin, carotenoids, CoQ10, and fat-soluble vitamins. The lipid phases in these systems facilitate aqueous dispersion and intestinal solubilization [188]. In contrast, protein-based nanoparticles and microgels provide stronger structural protection and are particularly useful for oxidation-sensitive compounds, peptides, and probiotics [75]. Hydrogel-based systems offer additional opportunities for controlling gastrointestinal residence time and digestion-triggered release, making them attractive for applications requiring temporally coordinated nutrient delivery [109].

The format of the product plays a crucial role in determining the suitability of a delivery system. Liquid sports nutrition products require low viscosity, high physical stability, rapid gastric emptying, and acceptable sensory properties [140]. Under these conditions, highly structured delivery systems may be difficult to implement despite their favorable encapsulation performance. On the other hand, semi-solid gels and recovery supplements can accommodate more complex microstructures and controlled-release mechanisms [11]. Solid products such as protein bars further allow delivery systems to become integrated into the food matrix itself, enabling matrix-mediated control of digestion and nutrient release [180].

The optimal delivery strategy may also vary according to the timing of nutrient consumption. Before exercise, delivery systems are often expected to provide sustained nutrient availability while minimizing gastrointestinal discomfort [157]. During exercise, factors like ease of rapid ingestion, hydration compatibility, osmotic control, and gastrointestinal tolerance become more critical [139]. After exercise, the rapid availability of amino acids, antioxidants, and recovery-related bioactives becomes more important than prolonged retention [149]. Consequently, delivery systems that perform well in one exercise stage may not necessarily be ideal for another.

Taken together, current evidence suggests that the selection of delivery systems in sports nutrition should not be based solely on encapsulation efficiency, structural stability, or release rate. Instead, delivery design should be guided by the interactions among bioactive characteristics, food matrix properties, intended consumption scenario, and physiological objectives. Although different delivery strategies exhibit distinct advantages under specific sports nutrition conditions, several important scientific and engineering challenges continue to limit their broader implementation in real products. These challenges are discussed in the following section.

## 5. Current Challenges and Future Design Directions

**Figure 5:**
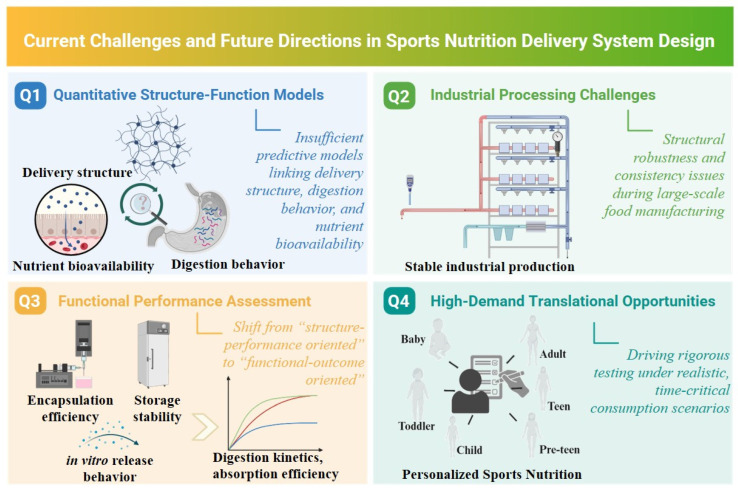
Current challenges and future directions in the design of food-grade delivery systems functional foods. Major priorities include establishing predictive structure–digestion–function relationships, improving structural robustness during industrial processing, developing function-oriented evaluation frameworks, and advancing application-driven design under realistic consumption conditions.

Although food-grade delivery systems have made substantial advances in both design concepts and structural formats, their practical translation into sports nutrition products still faces a series of unresolved scientific and engineering barriers. These challenges not only constrain the predictability of delivery performance but also hinder robust implementation in real sports nutrition formulations (as shown in Figure 5).

### 5.1. Lack of Quantitative Links Among Structure, Digestion Behavior, and Bioavailability

Currently, the design of food delivery systems relies heavily on empirical methods, especially when it comes to balancing structural stability with functional release. While there is increasing evidence that the architecture of these delivery systems can affect digestion rates and the release of bioactive compounds, systematic quantitative relationships between structural attributes—such as size, compactness, and interfacial composition—digestion behavior, and final bioavailability have not been well established [169]. This knowledge gap makes it difficult to transition from qualitative intuition to rational prediction, where “structural parameters” can be effectively translated into “functional outcomes.”

This issue is especially critical in sports nutrition, where post-exercise replenishment is time-sensitive. Many existing systems exhibit release trends primarily observed in in vitro digestion models; however, these findings often fall short of adequately predicting absorption efficiency in real physiological conditions related to exercise and recovery [189]. Therefore, future research should focus on integrating structural characterization, dynamic in vitro digestion kinetics, and function-relevant endpoints to develop predictive structure–function models that offer improved translational value.

### 5.2. Structural Robustness and Batch-to-Batch Consistency During Industrial Scale-Up

Transitioning food-grade delivery systems from laboratory-scale preparation to industrial manufacturing remains one of the major barriers to practical implementation. While many delivery structures can be reproducibly fabricated under controlled laboratory conditions through precise regulation of pH, shear intensity, component ratios, and assembly pathways, industrial food production introduces substantially greater variability. Factors such as differences in thermal treatment, homogenization pressure, mixing sequence, residence time, cooling rate, and raw material composition can all influence the formation and stability of delivery structures, ultimately affecting their functional performance [190,191].

Importantly, scaling up production is not simply a matter of increasing volume. Many food-grade delivery systems, particularly protein nanogels, protein–polysaccharide hydrogels, emulsion gels, and self-assembled colloidal structures, rely on finely balanced intermolecular interactions and interfacial organization. These structural features are often highly sensitive to processing history. During large-scale manufacturing, variations in shear forces, thermal exposure, and ingredient heterogeneity may alter particle size distribution, interfacial thickness, gel network architecture, and encapsulation microenvironments [192]. Consequently, delivery systems that exhibit excellent encapsulation efficiency and digestion performance at laboratory scale may experience aggregation, phase separation, premature leakage of bioactives, or altered digestion behavior after industrial processing.

This challenge is particularly relevant for sports nutrition products. Compared to conventional functional foods, sports nutrition products frequently contain relatively high concentrations of active ingredients, are consumed repeatedly over short periods, and require predictable physiological responses within specific exercise-related timeframes. Under these circumstances, even minor structural deviations arising during scale-up may result in noticeable differences in sensory perception, gastrointestinal tolerance, nutrient release kinetics, and overall functional efficacy [193]. Therefore, maintaining structural reproducibility across manufacturing batches becomes equally important as achieving high encapsulation efficiency.

For protein-based and gel-based delivery systems, several failure modes may emerge during scale-up. Excessive shear during industrial homogenization can disrupt self-assembled protein aggregates, weaken interfacial layers, or alter gel microstructures. Conversely, insufficient homogenization may result in broad particle size distributions and inconsistent release behavior [29]. Additionally, thermal treatments applied during pasteurization, sterilization, or drying may induce unintended protein denaturation, aggregation, or network restructuring, thereby affecting gel strength, colloidal stability, and digestion-triggered release characteristics [194]. Variability in raw material composition, especially for natural proteins and polysaccharides, may further exacerbate batch-to-batch inconsistencies.

Addressing these challenges requires a shift from formulation-centered optimization toward structure–process–function integration. Future development should focus on identifying critical quality attributes (CQAs) that govern delivery performance, including particle size distribution, zeta potential, interfacial composition, gel network density, encapsulation microenvironment, and digestion-triggered release kinetics [195]. At the same time, critical process parameters (CPPs) such as homogenization pressure, thermal history, mixing sequence, residence time, and cooling conditions, should be systematically linked to these structural attributes [196]. Establishing quantitative relationships among processing conditions, structural characteristics, and functional outcomes may provide a rational basis for designing delivery systems that remain robust across manufacturing scales.

Notably, despite significant advancements in food-grade delivery technologies, relatively few studies have successfully translated laboratory-scale proof-of-concept systems to pilot-scale or commercial sports nutrition products. Most published studies continue to focus on encapsulation efficiency, storage stability, and in vitro digestion performance under highly controlled experimental conditions. In contrast, systematic evaluations of processing-induced structural evolution, batch-to-batch consistency, long-term shelf-life robustness, and scale-dependent functional performance remain limited. Consequently, establishing predictive structure-process-function frameworks, along with pilot-scale validation studies under realistic manufacturing conditions, should be a top priority for future research.

### 5.3. Mismatch Between Current Evaluation Metrics and Real Functional Performance

The current evaluation of food delivery systems primarily focuses on metrics such as encapsulation efficiency, storage stability, and in vitro release behavior [197]. While these indicators are useful for early-stage screening, they often do not reflect functional outcomes in realistic sports nutrition scenarios. For example, a high encapsulation efficiency does not necessarily indicate high absorption efficiency. Additionally, structures that demonstrate high stability in vitro may have limited disassembly and release during digestion [198].

In the context of sports nutrition, the effectiveness of a delivery system is determined by its ability to enhance nutrient absorption within the appropriate time frame, while minimizing gastrointestinal burden and supporting intended physiological functions [199]. Therefore, assessment frameworks should transition from being “structure-performance oriented” to “functional-outcome oriented.” This transition should involve the integration of metrics related to digestion kinetics, absorption efficiency, and the specific needs of post-exercise recovery.

### 5.4. Sports Nutrition as a Key Translational Driver for Rational Delivery Design

Despite these challenges, sports nutrition also provides a distinctive opportunity for advancing food delivery science. As a high-load, high-frequency, and time-sensitive field, sports nutrition has specific performance requirements that can drive the transition of delivery systems from random configurations to systematic designs [200].

By continually validating and refining the relationships between structure and function in this demanding context, we may be able to develop more workable and widely applicable design frameworks. This progress would not only improve delivery applications in sports nutrition but could also lead to wider applications in various functional food systems [201]. From a translational perspective, patients with chronic liver disease are a key population for testing these delivery systems due to their well-known malabsorption profiles and urgent need for effective nutritional support.

## 6. Conclusions

This review emphasizes the growing importance of food-grade delivery systems in sports nutrition and highlights the necessity of evaluating their functional performance from a design-driven perspective rather than just a material-driven one. In sports nutrition applications, delivery systems must meet several criteria simultaneously: stable processing, sensory compatibility, responsiveness in the gastrointestinal tract, and effective nutrient release. Consequently, structural design is key to determining functionality. Current research suggests that protein-based and multi-component delivery structures offer significant advantages when incorporating bioactive compounds into liquid, solid, and semi-solid sports nutrition products. In particular, responsive and digestion-triggered systems hold great promise for enhancing gastric tolerance while ensuring efficient intestinal absorption by leveraging inherent gastrointestinal conditions.

However, there are considerable challenges that remain. These include the absence of predictable relationships among structure, digestion behavior, and functional outcomes, limited durability during industrial scale-up, and evaluation methods that do not accurately reflect real nutritional performance. Therefore, future research should focus on integrating structural characterization, digestion modeling, and function-oriented assessments to transition from empirical formulations to rational design. Overall, sports nutrition serves not only as a critical application area for food-grade delivery systems but also as a valuable framework for validating how food structure influences nutrient delivery and functional performance in real consumption scenarios.

## Figures and Tables

**Table 1:** Comparative overview of food-grade delivery systems for representative bioactive compounds in sports nutrition applications.

Bioactive Type	Representative Compounds	Major Instability/Delivery Challenge	Representative Delivery System	Structural Characteristics	Characteristics Key Advantages	Refs
Hydrophobic bioactives	Curcumin	Extremely low water solubility; oxidation sensitivity; poor intestinal absorption	Protein-stabilized nanoemulsion	Oil droplets stabilized by whey protein or plant protein interfaces	Improves aqueous dispersibility and intestinal bioaccessibility	[122]
Hydrophobic bioactives	Coenzyme Q10 (CoQ10)	Low water solubility and limited absorption	Protein–lipid emulsion system	Lipophilic core embedded within protein-coated lipid droplets	Improved lipid-phase incorporation and intestinal transport	[123]
Hydrophobic bioactives	Carotenoids (β-carotene, lutein)	Oxidative degradation and poor bioavailability	Nanoemulsion/multilayer emulsion	Small droplet size with multilayer interfacial protection	Enhanced oxidative stability and controlled digestion	[124]
Oxidation-sensitive compounds	Omega-3 fatty acids (EPA/DHA)	Lipid oxidation; unpleasant flavor; instability during processing	Protein–polysaccharide emulsion	Multilayer interfacial network formed by protein and polysaccharide interactions	Improved oxidative protection and emulsion stability	[125]
Oxidation-sensitive compounds	Polyphenols (catechins, resveratrol)	Oxidation and interaction with food matrices	Protein microgels	Crosslinked protein networks entrapping bioactives	Improved stability under acidic and thermal conditions	[126]
Hydrophilic bioactives	Branched-chain amino acids (BCAAs)	Rapid dissolution and fast gastric emptying	Hydrogel-based delivery systems	Water-rich polymeric network with embedded nutrients	Controlled gastric residence and gradual intestinal release	[127]
Hydrophilic bioactives	Glutamine	Instability during processing and storage	Protein–carbohydrate matrix	Nutrients physically entrapped within composite matrix	Enhanced stability in solid formulations	[128]
Hydrophilic bioactives	Caffeine	Bitter taste; rapid absorption; gastric irritation risk	Filled hydrogel or emulsion gel	Encapsulation within structured aqueous network	Taste masking and moderated release	[129]
Protein-derived bioactives	Bioactive peptides	Enzymatic degradation and instability during digestion	Protein self-assembled nanoparticles	Protein aggregates formed through heat or pH-induced assembly	Improved protection during gastric digestion	[130]
Lipophilic vitamins	Vitamin D	Low water solubility and oxidation sensitivity	Protein-stabilized nanoemulsion	Lipid-core droplets stabilized by food proteins	Improved incorporation into aqueous beverages	[131]
Lipophilic vitamins	Vitamin E	Oxidation and poor dispersion	Emulsion-filled hydrogel	Lipid droplets physically confined within gel matrix	Combined oxidative protection and controlled release	[132]
Multi-functional bioactives	Curcumin + Omega-3 co-delivery	Different polarity and stability requirements	Multicomponent hybrid delivery system	Protein–lipid–polysaccharide composite structure	Simultaneous stabilization of multiple bioactives	[133]

Notes: EPA, eicosapentaenoic acid; DHA, docosahexaenoic acid; BCAAs, branched-chain amino acids.

**Table 2:** Representative formulation components, functional ingredients, and physicochemical constraints in liquid sports nutrition systems.

Category	Representative Component	Common Concentration Range	Physiological Rationale	Formulation Constraint	Refs
Base carbohydrate sources	Glucose	Part of total carbohydrates (6–8%, *w*/*v*)	Rapid intestinal absorption via SGLT1; supports immediate energy supply during exercise.	High solubility required; excessive concentration increases osmolality and delays gastric emptying.	[139,140,141]
	Fructose	Often combined with glucose (≈1:0.5–1 ratio)	Absorbed via GLUT5; enables higher total carbohydrate uptake when combined with glucose.	Limited tolerance at high doses; requires homogeneous dispersion to avoid gastrointestinal discomfort.	[139,141,142]
	Sucrose	Included as mixed carbohydrate source	Hydrolyzed into glucose and fructose, supporting dual-transporter absorption.	Contributes to sweetness and osmolality; limits additional encapsulated components.	[143,144]
	Maltodextrin (glucose polymers)	Variable DP (Degree of Polymerization); contributes to 6–8% total carbohydrates	Provides higher carbohydrate delivery without proportional increase in osmolality.	Favors low-viscosity formulations; constrains particle size and phase separation of delivery structures.	[145,146]
Electrolytes	Sodium (Na^+^)	~20–50 mmol·L^−1^	Maintains fluid balance; enhances glucose and water absorption; prevents hyponatremia.	Ionic strength may destabilize protein- or polysaccharide-based delivery systems.	[144,147]
	Potassium (K^+^)	Typically < 10 mmol·L^−1^	Supports neuromuscular function and intracellular electrolyte balance.	Minor effect on structure but contributes to total ionic load.	[148]
Osmolality	/	~280–330 mOsm·kg^−1^	Optimizes gastric emptying and intestinal fluid absorption.	Strongly limits total solids, viscosity, and allowable encapsulation strategies.	[149,150]
Sports-related functional ingredients	Creatine	1–5 g per serving	Enhances phosphocreatine availability and high-intensity performance.	Low water solubility; limited stability in acidic solutions.	[151,152]
	HMB—β-hydroxy methylbutyrate	1–3 g per serving	Reduces muscle protein breakdown; supports recovery.	Bitter taste; usually formulated as salts.	[153,154]
	Caffeine	2–6 mg·kg^−1^ body mass	Improves alertness, endurance, and perceived exertion.	Dose-sensitive; GI tolerance and regulatory limits.	[151,155]
	Sodium bicarbonate	0.2–0.3 g·kg^−1^ body mass	Extracellular buffering; delays fatigue during high-intensity exercise.	High GI discomfort risk; poor palatability in liquids.	[156,157]
Flavoring/acidity	Organic acids (e.g., citric acid, malic acid, lactic acid, tartaric acid)	pH~3.0–4.0	Improves microbial stability and palatability.	Acidic environment may destabilize protein-based carriers during storage.	[158,159]
Viscosity/physical state	/	Low viscosity; Newtonian or weakly shear-thinning	Facilitates rapid ingestion and gastric emptying during exercise.	Restricts use of large particles, gels, or highly structured delivery systems.	[160,161]

Note: SGLT1, sodium-glucose cotransporter 1; GLUT5, glucose transporter 5; DP, degree of polymerization; GI, gastrointestinal; HMB, β-hydroxy β-methylbutyrate; Na^+^, sodium ion; K^+^, potassium ion. Glucose, fructose, sucrose, and maltodextrin are included as base carbohydrate sources and formulation constituents rather than bioactive compounds or functional bioactives.

**Table 3:** Delivery system functions in solid and semi-solid sports nutrition under realistic ingestion conditions.

Product Format	Representative Food Matrix	Delivery System Integration Mode	Structural Role During Mastication & Gastric Digestion	Release Behavior & Nutritional Implication	Key Design Constraint	Refs
Energy bar	Protein–carbohydrate composite matrix	Bioactives physically embedded within protein network	Matrix fragmentation during mastication governs particle size and gastric disintegration.	Gradual release driven by structural breakdown rather than dissolution.	Excessive matrix rigidity may limit digestion and bioaccessibility.	[166,176,177,178]
Protein bar	High-protein, low-moisture solid	Protein network acts as both nutrient and carrier	Chewing-induced fracture controls exposure of encapsulated compounds.	Delayed gastric release with extended intestinal availability.	Poor palatability and incomplete digestion if over-structured.	[178,179,180,181]
Sports gel	Viscoelastic hydrogel	Microgels or filled gel networks	Partial structural integrity maintained in stomach, followed by enzymatic loosening.	Phase-dependent release supporting staged nutrient delivery.	Narrow window between stability and gastric tolerance.	[182,183,184]
Chewable soft supplement	Soft solid or gummy matrix	Delivery system dispersed within deformable matrix	Mastication reduces size while preserving microstructure.	Moderated release linked to digestion rather than immediate dissolution.	Sensory perception of particulates.	[185,186,187]

## Data Availability

No data was used for the research described in the article.

## Supplementary Materials

The following supporting information can be downloaded at: https://www.mdpi.com/article/10.3390/gels12060525/s1, Figure S1. Flowchart of literature search and study selection.

## Abbreviations

The following abbreviations are used in this manuscript:

BCAAs	branched-chain amino acids
CoQ10	Coenzyme Q10
EPA	eicosapentaenoic acid
DHA	docosahexaenoic acid
SGLT1	sodium-glucose cotransporter 1
GLUT5	glucose transporter 5
DP	degree of polymerization
GI	gastrointestinal
HMB	β-hydroxy β-methylbutyrate
Na^+^	Sodium ion
K^+^	Potassium ion
