# Supplementary material for: Design-Driven Gel-Based Delivery Systems for Bioactives in Sports Nutrition
Source: Gels. 2026 Jun 11;12(6):525. doi: 10.3390/gels12060525 (PMC13298160; doi:10.3390/gels12060525)
Supplement: Supplementary file 1 [file gels-12-00525-s001.zip › gels-4356476-supplementary.pdf]

Supplementary Methods for Literature Collection and Scope Definition

This review was conducted as a narrative review supported by a structured literature search. The objective was to identify and synthesize studies relevant to the design of food-grade delivery systems for bioactive compounds, with particular emphasis on structural stability, digestion responsiveness, bioavailability-related performance, and applications in sports nutrition and precision nutrition products.

A comprehensive literature search was performed using major electronic databases, including Web of Science, Scopus, PubMed/Medline, ScienceDirect, and Google Scholar. The search covered publications from 2006 to 2026 and was restricted to articles written in English. The following keywords were used alone or in combination: “food delivery system”, “bioactive compounds”, “encapsulation”, “nanoencapsulation”, “protein-based delivery system”, “protein self-assembly”, “protein–polysaccharide complex”, “protein–lipid system”, “emulsion”, “hydrogel”, “microgel”, “digestion-responsive delivery”, “gastrointestinal digestion”, “bioaccessibility”, “bioavailability”, “sports nutrition”, “functional foods”, and “precision nutrition”. Additional relevant studies were identified by screening the reference lists of key reviews and highly relevant original articles.

The inclusion criteria were as follows: (i) original research articles, reviews, and systematic reviews related to food-grade delivery systems for bioactive compounds; (ii) studies addressing protein-based, polysaccharide-based, lipid-based, or multicomponent delivery structures; (iii) studies reporting processing stability, storage stability, gastrointestinal digestion behavior, bioaccessibility, bioavailability, or release performance; and (iv) studies relevant to functional foods, sports nutrition products, precision nutrition, or real food matrices. The exclusion criteria were: (i) studies focused exclusively on pharmaceutical or injectable delivery systems without relevance to food applications; (ii) studies lacking information on delivery structure, bioactive compounds, or food matrix applicability; (iii) studies not available in English; and (iv) conference abstracts, editorials, patents, and non-peer-reviewed materials unless they provided essential contextual information.

Article screening was conducted in two stages. First, titles and abstracts were screened to remove irrelevant records. Second, the full texts of potentially relevant articles were assessed according to the inclusion and exclusion criteria. The selected literature was then synthesized narratively rather than statistically, because the included studies differed substantially in delivery materials, bioactive compounds, food matrices, processing conditions, digestion models, and functional endpoints.

For each included study, the following information was extracted where available: delivery material, structural format, encapsulated bioactive compound, preparation strategy, food matrix, processing or storage stability, gastrointestinal digestion behavior, release characteristics, bioaccessibility or bioavailability-related outcomes, and potential application in sports or precision nutrition. The extracted information was organized around four major themes: core design principles, emerging delivery structures, real food applications, and current challenges and future design directions. A flowchart summarizing the literature selection process is provided in the **Figure S1**.

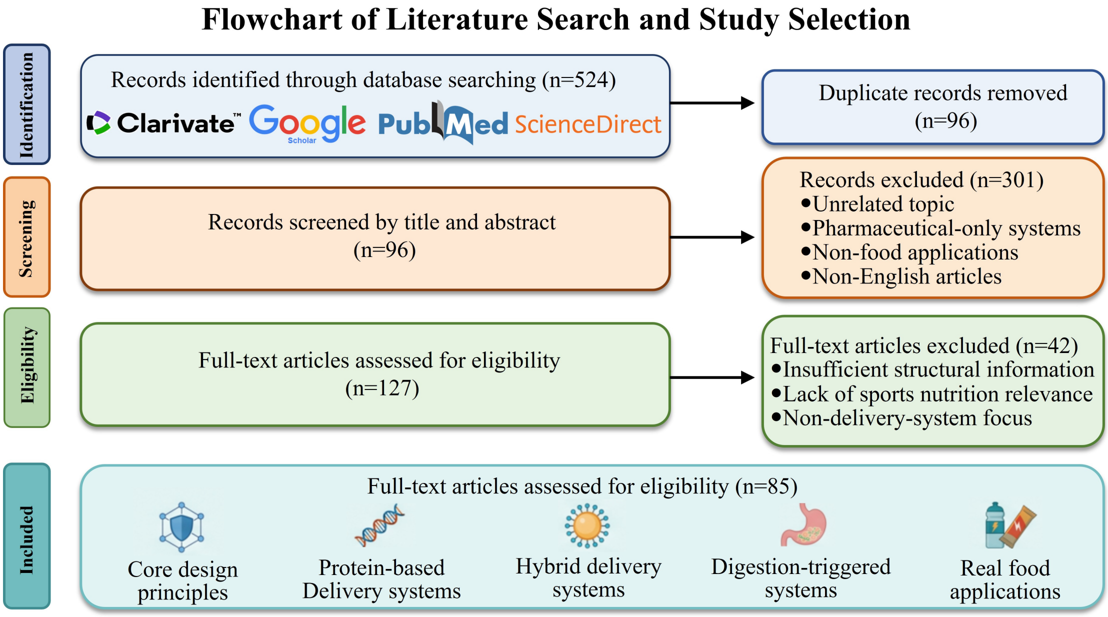

Figure S1. Flowchart of literature search and study selection.
